# Dry Mouth Dilemma: A Comprehensive Review of Xerostomia in Complete Denture Wearers

**DOI:** 10.7759/cureus.58564

**Published:** 2024-04-18

**Authors:** Swapnali Mhatre, Reema Srichand, Jyotsna Sethumadhavan, Pallavi B Mishra, Srushti D Patil, Riddhi S Chavan, Mridula Joshi, Uttam Shetty

**Affiliations:** 1 Department of Prosthodontics and Crown and Bridge, Dental College and Hospital, Bharati Vidyapeeth (Deemed to be University), Navi Mumbai, IND; 2 Medical School, Dental College and Hospital, Bharati Vidyapeeth (Deemed to be University), Navi Mumbai, IND

**Keywords:** oral lubricating devices, oral salivary substitute, hyposalivation, complete denture prosthesis, xerostomia

## Abstract

Xerostomia, commonly known as dry mouth, presents a significant challenge for individuals wearing complete dentures, affecting their oral health and quality of life. This review explores the relationship between saliva and complete dentures, highlighting the varied management strategies for xerostomia. Saliva plays a critical role in denture retention, lubrication, and oral environment buffering. Complete denture wearers often experience reduced salivary flow, aggravating symptoms of xerostomia. Various management approaches are discussed, including general measures such as hydration and salivary stimulation techniques which aim to boost saliva production naturally. The use of salivary substitutes provides artificial lubrication and moisture to alleviate dry mouth discomfort. Oral lubricating devices, such as sprays, gels, and lozenges, offer relief by mimicking saliva's lubricating properties, thereby improving denture stability and comfort. This review addresses the etiology of xerostomia in complete denture wearers and explores preventive measures to reduce its impact. A comprehensive approach has been discussed for the management of xerostomia which will help to improve the oral health and well-being of complete denture wearers experiencing dry mouth.

## Introduction and background

The presence of optimal salivary flow, consistency, and composition holds importance, particularly in completely edentulous patients. It is imperative for the prosthodontist to meticulously consider these salivary characteristics throughout the stages of denture fabrication from initial assessment to post-insertion care. Salivary flow is crucial for maintaining oral health and comfort. The average daily secretion of saliva typically ranges from 500 ml to 1500 ml (approximately 50.72 oz), underscoring its significance in oral function and comfort [1]. Xerostomia, characterized by dryness of the oral cavity, arises due to a reduction or absence of salivary flow. It is a symptomatic manifestation of multifactorial conditions such as radiation exposure or a side effect of certain medications [2].

Oral mucosal changes in xerostomia commonly occur with alterations in the appearance of the oral mucosa. The mucosa may exhibit a cobblestoned or erythematous appearance, resembling a pebbled texture due to reduced lubrication and moisture. In cases of chronic xerostomia, the filiform papillae on the dorsal surface of the tongue may undergo atrophy. This can result in a smooth, glossy appearance of the tongue surface, known as a "bald tongue" or "smooth tongue." Xerostomia can contribute to the development of fissures or cracks on the surface of the tongue (fissured tongue) which can be associated with discomfort and pain [2].

The oral mucosal surfaces may exhibit increased adherence or stickiness upon light palpation. This can occur due to insufficient lubrication provided by saliva, leading to a sensation of dryness and discomfort in the oral cavity [3]. Diagnostic tests such as cotton wool drying followed by salivary gland milking may reveal delayed or absent salivary secretion from the duct orifices. This delay in salivary flow further confirms the presence of xerostomia and underscores the need for prompt intervention and management [4]. These clinical signs collectively contribute to the diagnosis and assessment of xerostomia in patients.

The etiology of xerostomia

The etiology of xerostomia encompasses a broad spectrum of factors, primarily categorized into two groups, water or metabolic loss and salivary gland damage, along with interference with neural transmission. These causes have significant implications for the management and treatment of xerostomia. Water or metabolic loss may occur due to dehydration arising from various conditions such as pyrexia, burns, hyperhidrosis, hemorrhage, vomiting, diarrhea, and renal dysfunction causing excessive urination or osmotic polyuria [5]. Protein-calorie malnutrition can exacerbate fluid and electrolyte loss, further contributing to xerostomia. Salivary gland impairment plays a crucial role in decreasing saliva production due to therapeutic radiation targeting the head, neck, and face, which can lead to salivary gland dysfunction. Diseases affecting the salivary glands are autoimmune disorders like Sjögren's syndrome, endocrine conditions such as type I and II diabetes mellitus, thyroid disorders, and adrenal gland disorders, which affect their function and reduce saliva production [6].

Systemic diseases like liver and kidney diseases, including hepatitis C infection, as well as viral infections, such as human immunodeficiency virus (HIV) and human T-lymphotropic virus-1 (HTLV-1), can indirectly affect salivary gland function, contributing to xerostomia [7]. Medications interfering with the neural control of salivary glands, such as cytotoxic drugs, anticholinergic drugs, proton pump inhibitors, psychoactive agents, sympathomimetic drugs, antihypertensives, and diuretics, are associated with dry mouth as a side effect [8]. Other factors contributing to xerostomia include age-related medication usage. Tobacco use, whether through chewing or smoking, increases the risk of dry mouth by affecting saliva production and quality [9].

Dehydration, caused by insufficient fluid intake, can also contribute to dry mouth. Physical exertion or exposure to heat may lead to temporary dry mouth due to dehydration and prioritization of fluid distribution away from the salivary glands [10]. Various health conditions and habits can also contribute to xerostomia, including anxiety disorders, depression, Parkinson's disease, poorly managed diabetes, Sjögren's syndrome, and certain sleep habits like mouth breathing or snoring [11]. Stroke and Alzheimer's disease may cause a perception of dry mouth, even when salivary gland function is intact [12]. For management and treatment strategies specific to address the underlying causes and alleviate symptoms effectively, understanding the multifactorial etiology of xerostomia is essential.

Signs and symptoms

Dry mouth, or xerostomia, presents a range of signs and symptoms that can significantly impact oral health and quality of life. One of the primary symptoms associated with dry mouth is halitosis or bad breath which arises due to decreased salivary flow and the subsequent build-up of bacteria in the oral cavity [13]. Cheilitis, characterized by inflammation, splitting, and cracking of the lips, may also occur due to inadequate moisture and lubrication provided by saliva. Cracking and splitting of the oral mucosa in the cheeks and lips can lead to discomfort and pain, as the skin at the corners of the mouth is susceptible to soreness and ulceration [14]. Dry mouth often results in dysgeusia or taste disorders, affecting the perception of flavors and altered taste sensations. Reduced saliva flow predisposes individuals to fungal infections in the mouth, such as oral thrush, which can manifest as white patches or lesions on the tongue, inner cheeks, or palate [15]. Glossodynia may also occur due to dryness and irritation of the oral mucosa.

Inflammation of the tongue or the development of tongue ulcers may cause oral discomfort and compromise oral function. Dry mouth is associated with a higher incidence of periodontal disease, as saliva plays a crucial role in maintaining oral hygiene and buffering acidic pH levels in the mouth. Increased tooth decay and plaque accumulation are also common as saliva helps remineralize tooth enamel and neutralize acids produced by bacteria [16]. Practical difficulties in oral function are prevalent among individuals with dry mouth, including problems in speaking, swallowing, and chewing. Individuals wearing dentures may experience issues with denture retention, development of denture sores, and adhesion of the tongue to the roof of the mouth due to reduced saliva lubrication [17]. In severe cases of xerostomia, complications such as sialadenitis, a salivary gland infection, may arise, leading to localized pain, swelling, and inflammation of the affected gland [18]. Sore throat, sticky or stringy saliva, and compromised oral hygiene further contribute to the multifaceted nature of xerostomia's clinical presentation.

Drugs linked with dry mouth

Several classes of pharmaceuticals have been linked with xerostomia. Phenothiazines and benzodiazepines, commonly used for various psychiatric and neurological conditions, have been reported to cause dry mouth as a side effect [19]. Anticholinergic agents such as atropine and hyoscine, which block the action of acetylcholine in the body, can inhibit saliva secretion and contribute to xerostomia [20]. Opioids, including medications like morphine and codeine, are known to suppress saliva production through their central nervous system effects [21]. Certain cytotoxic drugs used in chemotherapy, which are intended to target rapidly dividing cells, can also damage salivary glands and impair saliva production [22].

Medications that influence the sympathetic nervous system can indirectly contribute to dry mouth by altering salivary gland function [23]. Acid pump inhibitors, including pantoprazole, commonly used to treat gastroesophageal reflux disease (GERD) and peptic ulcers, have also been associated with xerostomia as a potential adverse effect [24]. Alpha-1 antagonists like doxazosin, beta blockers like carvedilol, and alpha-2 agonists such as clonidine have all been reported to induce xerostomia. Diuretics subsequently reduce fluid levels in the body and can lead to dehydration and dry mouth [25]. Centrally acting psychoactive drugs, including certain antidepressants such as tricyclic compounds, have anticholinergic effects that can interfere with saliva production [26].

Consequences and complications

Xerostomia can have wide-ranging consequences and complications that impact oral health, comfort, and overall well-being. Oropharyngeal dysfunction, including dysphagia, can significantly impact an individual's ability to eat and drink comfortably [27]. Dysarthria can affect communication and social interaction [28]. It can also cause mucosal trauma, ulcerations, and pharyngoesophageal pain [29]. Reduced saliva flow contributes to mucus accumulation in the mouth and difficulty in clearing the throat. Food residues may also accumulate in the oral cavity due to inadequate saliva for lubrication and cleansing, increasing the risk of plaque formation and dental caries. Hyposalivation compromises the mouth's natural defense mechanisms against bacterial overgrowth, resulting in alterations in the oral microflora and an increased susceptibility to oral infections such as candidiasis [30]. Nocturnal oral discomfort due to dryness can disrupt sleep patterns and reduce overall quality of life [31].

## Review

Saliva and complete denture

Saliva also plays a very important role in preserving denture integrity by keeping the denture surfaces clean and in maintaining oral hygiene. It physically washes away food and other debris. The lubrication provided by saliva is important in the edentulous as this makes the surface of the dentures more compatible with the movements of the lips, cheek, and tongue during speech, mastication, and swallowing. Salivary β-glycoproteins facilitate the movement of soft tissues during oral functions and contribute to denture retention. Successful rehabilitation of edentulous patients with complete dentures has largely contributed to satisfactory denture retention [32]. The physical factors contribute to denture retention, including adhesion, cohesion, interfacial surface tension, atmospheric pressure, capillary attraction, and viscosity of saliva. Principal factors that aid denture retention are the adhesive action of the thin film between the saliva and denture base and underlying soft tissues. Such adhesive action of the saliva is achieved through ionic forces between charged salivary glycoproteins and surface epithelium on one side and denture base acrylic resin on the other. It also provides a cushioning effect and lubrication between the denture base and oral tissues and tends to reduce friction [33].

The quantity, quality, consistency, and flow rate affect the stability of the denture. A copious amount of thin viscosity saliva lubricates the mucosa and assists in denture retention. Dry mouth affects the retention of the denture and also leads to soft tissue trauma due to enhanced friction between the mucosa and denture. The presence of thick ropy saliva leads to dislodgement of the maxillary denture due to the negative hydrostatic force that is created in the anterior to posterior palatal seal. While taking impressions, tissue details are poorly recorded due to thick saliva. In excessive salivation, impression-making becomes difficult and may cause pits and voids.

Management of xerostomia

General Measures

The management of xerostomia and hyposalivation depends upon its etiology. A multidisciplinary approach allows for comprehensive care addressing both the oral and systemic aspects of the condition. Medication-related xerostomia can be reduced by substituting it with other medication with fewer side effects affecting salivary flow. Adjusting the timing or dosing schedule of medications can also help minimize dry mouth symptoms while maintaining therapeutic efficacy [2]. Hydration is essential, especially for individuals on low-salt diets or those using antidiuretic drugs, which can exacerbate dehydration. Patients are advised to consume at least 2 liters of fluid daily to ensure proper hydration and support salivary gland function. Multidisciplinary management of systemic conditions such as Sjögren's syndrome and other diseases like rheumatoid arthritis, HIV infection, and diabetes is necessary, which can contribute to xerostomia [34].

Salivary Stimulation

Salivary stimulation therapies offer effective options for managing xerostomia by enhancing saliva production and improving oral hydration. Stimulation methods can be achieved through mechanical or chemical means, often involving lifestyle modifications or pharmacological interventions. Mechanical stimulation involves strategies such as consuming more frequent meals and chewing sugar-free gum. Chewing gum stimulates saliva production by mechanically activating the salivary glands, promoting the release of saliva into the oral cavity. Xylitol, a sugar alcohol commonly used in chewing gum, has been shown to enhance saliva production and improve oral hydration [35].

Chemical stimulation, on the other hand, involves the use of sialagogues-medications that directly stimulate the salivary glands to increase saliva production. Several pharmacological agents have been identified for this purpose.

Pilocarpine: It is a cholinergic agonist that stimulates muscarinic receptors on the salivary gland cells, leading to increased saliva secretion. Pilocarpine has been extensively studied and shown to be effective in improving salivary flow rates and relieving symptoms of xerostomia [36].

Bromhexine: This mucolytic agent has been found to possess sialogogue properties by stimulating the secretion of saliva. It acts by enhancing the production and secretion of mucus in the salivary glands, thereby promoting saliva flow [37].

Cevimeline: Another cholinergic agonist that selectively activates muscarinic receptors in the salivary glands, resulting in increased saliva production. Cevimeline has been approved for the treatment of xerostomia associated with Sjögren's syndrome and has demonstrated efficacy in improving salivary flow rates and relieving dry mouth symptoms [2].

Anethole trithione: A compound with sialogogue properties, anethole trithione acts by stimulating saliva secretion through its cholinergic activity. It has been used in the treatment of xerostomia, although further research is needed to fully elucidate its efficacy and safety profile [38].

The duration of action for these sialagogues typically ranges from two to three hours, providing temporary relief from dry mouth symptoms. However, it is important to note that these medications may have side effects and contraindications and their use should be carefully monitored [2].

Use of Salivary Substitutes

The main drawback of artificial saliva is that patients need to manually introduce it into the oral cavity at regular intervals. The use of salivary substitutes is a key component of the treatment approach for xerostomia, aiming to reduce dry mouth symptoms and maintain oral health. Salivary substitutes can be categorized into various types, each with its own advantages and considerations.

Glycerin and lemon-based substitutes: These substitutes are relatively simple and readily available. They may have drawbacks such as potential erosion of natural teeth due to acidity and astringent properties and possible discomfort or stinging sensation in soft tissues due to glycerin content.

Carboxymethyl cellulose (CMC)-based substitutes: CMC-based substitutes offer a more complex formulation, providing improved lubrication and moisture retention compared to glycerin-based options. These substitutes can effectively coat oral surfaces and provide relief from dry mouth symptoms [2].

Mucin-based substitutes: Mucin is a glycoprotein present in natural saliva and contributes to its lubricating and moisturizing effects and protective qualities. Mucin-based substitutes that closely resemble the composition of natural saliva are considered to have superior properties in terms of mimicking natural saliva.

In addition to conventional artificial saliva products, milk can also serve as a natural salivary substitute due to its chemical and physical properties. Milk not only moisturizes and lubricates the oral mucosa but also helps buffer oral acidity, decrease enamel solubility, and promote enamel remineralization [39]. Despite the benefits of salivary substitutes, one drawback is the need for manual introduction into the oral cavity at regular intervals, as the effects are not sustained over extended periods. Patients may need to frequently administer these substitutes to maintain oral comfort and prevent complications.

Use of Oral Lubricating Devices

The utilization of oral lubricating devices presents an innovative approach to the treatment of xerostomia, offering a convenient and effective means of delivering saliva substitutes to reduce dry mouth symptoms and enhance oral comfort. These devices are designed to meet specific requirements to ensure optimal performance and patient satisfaction.

Reservoir Dentures

Reservoir dentures feature compartments or reservoirs within the denture base that can hold saliva substitutes or lubricants. These reservoirs gradually release the solution throughout the day, providing continuous relief from dry mouth symptoms [2]. Designing reservoirs in dentures involves a meticulous process aimed at integrating specialized compartments within the denture base to store and dispense salivary substitutes. A comprehensive assessment of the patient's oral health status, including the severity of xerostomia, oral tissue condition, and denture requirements, is crucial before designing reservoirs in dentures [40]. Patient evaluation should encompass factors such as the etiology of xerostomia, salivary gland function, and individual treatment objectives. Salivary gland function tests are diagnostic procedures used to assess the production and secretion of saliva by the salivary glands such as salivary flow rate measurement, sialometry, salivary pH test, salivary composition analysis, salivary gland imaging, and salivary gland biopsy.

**Figure 1:**
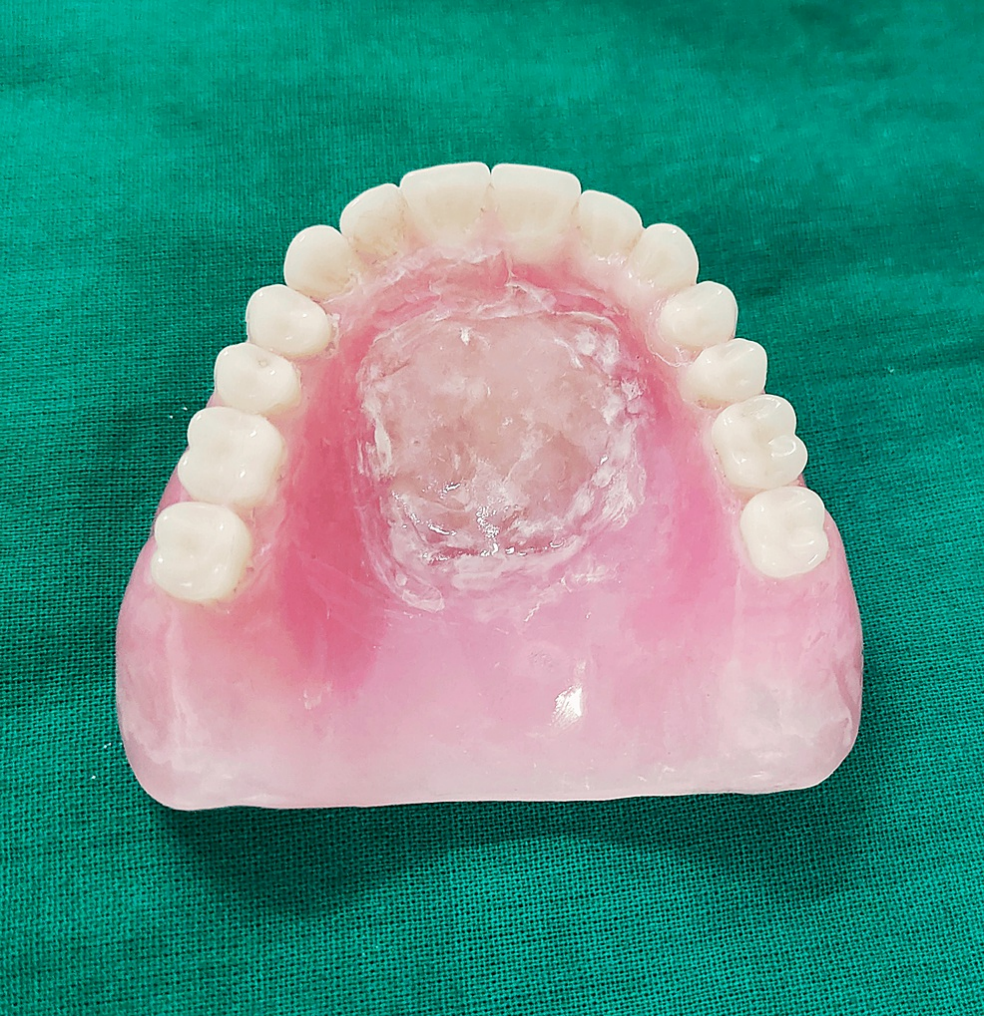
Maxillary denture with salivary reservoir

**Figure 2:**
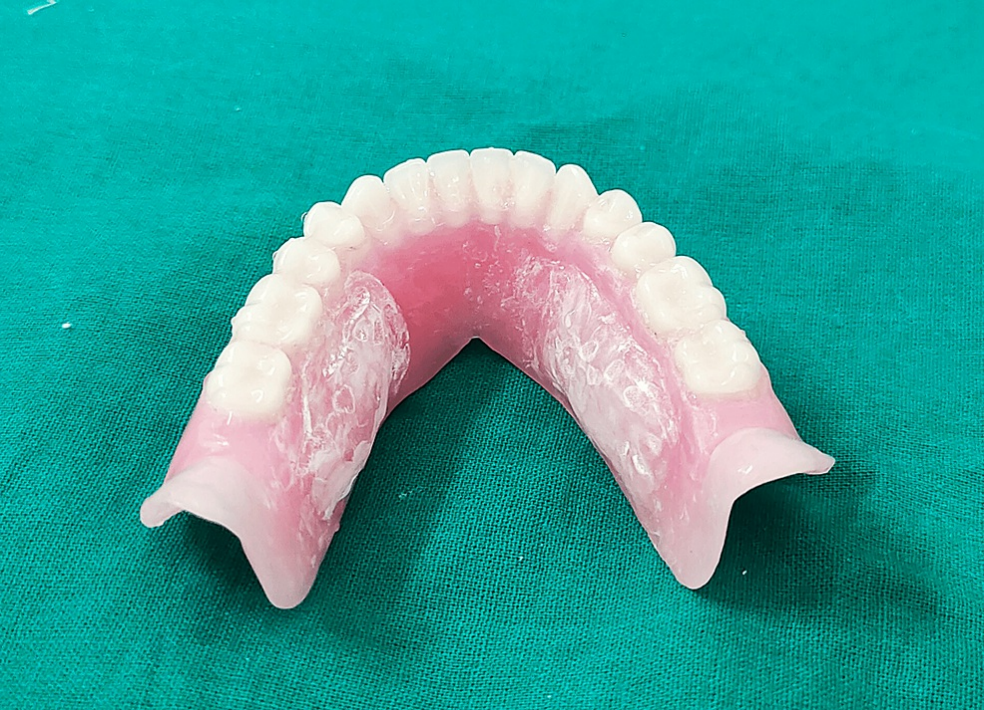
Mandibular denture with salivary reservoir

The placement and configuration of reservoirs within the denture base are important aspects of the designing process [41] for the optimal distribution and retention of saliva substitutes. The most common locations for reservoirs include the palate and the lingual aspect of the denture base. Through the electronic search of the keyword "salivary reservoir denture," 22 case series were found from 1986 to 2024 [42,43]. The location for reservoir preference seemed almost equal among both the arches, out of which, maxillary reservoirs were placed in the palate. Mandibular reservoirs were designed in the lingual flange or split denture technique with inter-compartments (Figure 1 and Figure 2).

Reservoirs in these areas ensure that saliva substitutes are evenly dispersed across the oral cavity, providing maximum relief from dry mouth symptoms. Reservoirs are designed to accommodate 2-5 ml volume of saliva substitutes; the duration of flow ranges from two to five hours while maintaining the integrity and aesthetics of the denture [44]. The shape of reservoirs may vary depending on the patient's oral anatomy and the desired distribution of saliva substitutes. Reservoirs may be elongated to cover a larger surface area of the palate or lingual tissues. The orientation of reservoirs is adjusted to optimize the release of saliva substitutes during mastication and speaking.

The choice of materials for reservoir fabrication should prioritize durability, flexibility, and biocompatibility with saliva substitutes. Medical-grade polymers, such as silicone elastomers, are commonly used [45]. These materials ensure that reservoirs remain comfortable and safe for long-term use, minimizing the risk of irritation or allergic reactions in the oral cavity. Saliva substitutes, such as artificial saliva or lubricating agents, are introduced into the reservoirs to provide moisture and lubrication to the oral mucosa [46]. Careful calibration of the reservoirs ensures the controlled release of saliva substitutes, preventing over-hydration or under-hydration of the oral tissues. Following the fabrication of dentures with reservoirs, thorough evaluation and patient feedback are essential to assess fit, comfort, and efficacy [47]. Any necessary adjustments to the reservoir design or placement are made based on patient response and clinical outcomes, ensuring optimal performance and patient satisfaction.

Other Oral Lubricating Devices

These devices are designed to administer saliva substitutes or lubricants, commonly induced by medication side effects, systemic conditions, or radiation therapy. Developed for sustained release, they maintain optimal moisture levels over time, ensuring enduring comfort for patients. With user-friendly designs, they allow for effortless application and administration, promoting patient compliance and convenience. Seamlessly integrating into the oral cavity, these devices do not interfere with normal oral function, enabling patients to speak, chew, and swallow comfortably while benefiting from their use.

Saliva sprays: Saliva sprays are portable and convenient devices that deliver a fine mist of artificial saliva directly into the mouth. These sprays offer quick relief, are suitable for on-the-go use, and offer immediate moisture to the oral cavity [36].

Oral gels: Oral gels are viscous formulations applied directly to the oral mucosa. They coat the tissues, providing lubrication and moisture to alleviate dryness and discomfort. These gels often contain ingredients such as glycerin, xylitol, or cellulose derivatives which help to retain moisture and soothe dry mouth symptoms [48].

Lozenges: Lozenges are solid formulations that dissolve slowly in the mouth, releasing active ingredients to moisturize and lubricate the oral tissues. They offer prolonged relief and are available in various flavors [49].

Mouth rinses: Some mouth rinses are specifically formulated to combat dry mouth symptoms. These rinses contain moisturizing agents such as glycerin or xylitol, which help to hydrate the oral tissues and alleviate dryness. Additionally, mouth rinses may have antimicrobial properties to promote oral health and prevent complications associated with dry mouth [50].

Oral lubricating devices work through various mechanisms to provide relief from dry mouth symptoms. The primary function of these devices is to replenish moisture in the oral cavity. Ingredients such as water, glycerin, or xylitol mimic the hydrating properties of saliva, helping to moisten the oral mucosa and alleviate dryness. Lubricating agents in oral gels, sprays, and lozenges coat the oral tissues, reducing friction and discomfort associated with dry mouth. This lubrication enhances oral comfort during speaking, chewing, and swallowing. Some oral lubricating devices contain ingredients that can stimulate salivary gland function, promoting the production of natural saliva. For example, xylitol-based products may stimulate saliva secretion by activating salivary reflexes. Certain formulations are designed to maintain the pH balance of the oral cavity, which is essential for oral health. By buffering acidic conditions, these devices help prevent oral mucosal irritation and protect against dental erosion. Oral lubricating devices contain antimicrobial agents that help reduce the risk of oral infections. These agents inhibit the growth of bacteria and fungi in the mouth, promoting oral hygiene and preventing complications associated with dry mouth.

Denture design modifications

Modified denture designs for patients with xerostomia involve specific alterations in both the design and fabrication process to address the challenges associated with reduced salivary flow and dry mouth symptoms. These modifications aim to enhance comfort, retention, and oral health for individuals experiencing xerostomia. One key aspect of modified denture design is the incorporation of reservoirs or compartments within the denture base. Softer and more flexible materials may be preferred to minimize friction and irritation on the oral mucosa. These materials offer greater comfort and reduce the risk of tissue trauma, especially in individuals with sensitive oral tissues [51]. Liquid-supported denture is a viable surface modification for xerostomic patients. The technique involves the incorporation of a polyurethane sheet in the denture to create space which was further filled with glycerin or any other lubricating liquid. This will help in reducing occlusal pressure on the ridge and soft tissues by the distribution of stresses through the liquid. This has the advantages of both denture relining material and soft liners which increases comfortability over long-term usage of the denture [52].

Surface modifications are another consideration in modified denture design for xerostomia patients. Special coatings or treatments such as soft liners may be applied to the denture surface to reduce friction and pressure spots on soft tissue. Soft liners acrylic or silicone type can be used depending on the shelf-life of the material. These surface modifications help improve the overall comfort and adaptability of the dentures, particularly in individuals with compromised salivary flow [53].

The contour and fit of the dentures are customized to accommodate changes in oral anatomy resulting from xerostomia. Prosthodontists pay close attention to the fit of the denture borders and ensure optimal retention and stability. This meticulous adjustment of the denture contours helps prevent displacement and discomfort during speaking and eating. Advanced techniques such as digital denture design and computer-aided design/computer-aided manufacturing (CAD/CAM) may be utilized to achieve accurate and predictable results [54].

Denture adhesives

It is seen that the wetting mechanism of saliva is very important for the retention of the complete denture. Denture adhesives have shown quite impressive results in the case of dry mouth. Denture adhesives come in various forms, including pastes, powders, and strips. The most common components of denture adhesives include polymers such as CMC or methyl cellulose, along with mineral oil, petrolatum, or other petroleum-based compounds. These ingredients work together to enhance adhesion between the denture and oral tissues.

Polymer-based adhesives, such as those containing CMC, form a gel-like layer when hydrated, which adheres to both the denture and the oral tissues. This adhesive layer fills in gaps and irregularities, improving the fit and stability of the denture. Mineral oil and petrolatum act as lubricants, reducing friction between the denture and the oral mucosa, thereby minimizing irritation and discomfort. Some denture adhesives may contain antimicrobial agents, such as zinc or fluoride, which help maintain oral hygiene by inhibiting the growth of bacteria and fungi on the denture surface. These antimicrobial properties contribute to overall oral health and may prevent oral infections associated with prolonged denture wear [55]. When selecting a denture adhesive for patients with xerostomia, it is essential to consider factors such as product compatibility, ease of application, and patient preference. Patients should be instructed to use an adequate amount of denture adhesives as per the manufacturer's instructions. Dentures with adhesives need to be wetted before use to maximize their benefits [56]. Maximum 12-14 hours denture adhesives can be used. Dentures need to be cleaned thoroughly before re-application to remove any remnants of old adhesive, which can cause bacterial and fungal growth.

Preventive measures

For the prevention of oral complications such as reduced salivary output, additional measures should be implemented, for example, dental evaluations should be carried out once every 4-6 months. Annual radiographs are recommended to assess for potential dental caries, bone loss, or other pathology [56]. To prevent dental caries, especially in patients wearing single complete dentures or overdentures, maintaining a low-sugar diet is advisable. Proper oral hygiene practices, including regular brushing and flossing, are essential. Furthermore, the use of topical fluoride treatments is recommended to strengthen tooth enamel and prevent decay. In cases of rampant caries due to hyposalivation, neutral pH sodium fluoride is effective [57]. Dentifrices, varnishes, oral gels, and mouth rinses containing fluorides and remineralizing solutions can be used with or without applicator trays. The specific fluoride regimen should be decided on the individual patient's needs, the magnitude of hypofunction of the salivary gland, and the rate of caries [58]. For patients undergoing radiation therapy, strategies to mitigate radiation-induced salivary dysfunction without compromising oncologic treatment are crucial. These may include sparing the parotid glands from radiation, using tissue protectants, and surgical transfer of salivary glands. These approaches aim to preserve salivary gland function and minimize the impact of radiation therapy on saliva production [59]. In denture-wearing patients, it is advised to wet dentures before placing them into the mouth and spraying prostheses with artificial saliva before applying denture adhesives as this will help in reducing the discomfort. Additionally, increasing fluid intake during meals and wetting dentures before eating can aid in mastication and swallowing, facilitating a more comfortable eating experience [2].

Advanced treatment for xerostomia

Electrical Stimulation

Electrical stimulation of salivary glands is a promising therapeutic approach for xerostomia. This technique involves the application of electrical currents to the salivary glands to stimulate saliva production. Electrical stimulation can enhance salivary gland function by increasing blood flow, promoting glandular cell proliferation, and stimulating neurotransmitter release [60]. Several studies have demonstrated the efficacy of electrical stimulation in improving saliva flow rates and relieving xerostomia symptoms in patients with various underlying conditions, including Sjögren's syndrome, radiation-induced xerostomia, and salivary gland hypofunction [61,62]. Electrical stimulation devices, such as intraoral electrostimulators, have shown promising results in clinical trials, providing a non-invasive and potentially effective treatment option for xerostomia [63]. These devices can be easily incorporated into the dentures or mouth guards to effectively and locally provide salivary stimulation.

Tissue Engineering

Tissue engineering approaches hold great promise for regenerating damaged or dysfunctional salivary glands in patients with xerostomia. Tissue engineering involves the construction of artificial tissues or organs using a combination of cells, biomaterials, and bioactive factors. In the context of xerostomia treatment, tissue engineering strategies aim to create functional salivary gland tissues capable of producing saliva [64]. Researchers have explored various approaches to tissue engineering for salivary glands, including the use of stem cells, bioengineered scaffolds, growth factors, and tissue culture techniques. By harnessing the regenerative potential of stem cells and the biocompatibility of biomaterials, tissue engineering holds the potential to restore salivary gland function and alleviate xerostomia symptoms [65]. Clinical studies evaluating tissue-engineered salivary gland constructs have shown promising results in preclinical models, demonstrating the feasibility of this approach for xerostomia treatment. However, further research is needed to optimize tissue engineering strategies and translate them into clinically viable treatments for xerostomia [66].

Gene Therapy

Gene therapy offers another innovative approach for treating xerostomia by targeting the underlying genetic or molecular causes of salivary gland dysfunction. Gene therapy involves the delivery of therapeutic genes to target cells or tissues to correct genetic defects, enhance cell function, or modulate gene expression [67]. In the context of xerostomia, gene therapy can be used to promote salivary gland regeneration, enhance saliva production, or protect salivary gland tissues from damage. Researchers have investigated various gene therapy approaches for xerostomia, including the delivery of growth factors, anti-inflammatory cytokines, or transcription factors to promote salivary gland function and repair. Preclinical studies using animal models have shown promising results for gene therapy in restoring salivary gland function and alleviating xerostomia symptoms. However, the clinical translation of gene therapy for xerostomia remains in the early stages, with further research needed to optimize gene delivery methods, ensure safety and efficacy, and address potential regulatory challenges [62].

## Conclusions

Xerostomia is a condition which affects the overall health and quality of life. The above review summarises the challenges faced by a xerostomic denture patient and measures which can be introduced to overcome them. The xerostomic patient faces not only difficulties due to decreased salivary flow but also increased chances of trauma to mucosal surfaces. The review has briefed about xerostomia in detail including etiology, characteristics, and management. The ways to reduce the impact of dry mouth include general measures along with systemic drugs used at times in conjunction with salivary stimulation. Various denture surface modifications have been incorporated to lessen the struggle in adaptation to dentures. Salivary substitutes also play a vital role in the efficient management of xerostomia. Similarly, tissue engineering, gene therapy, and electric stimulation represent an innovative approach to enhance salivary gland function. However, further research and clinical trials are needed to validate their efficacy, safety, and long-term outcomes in human patients.
